# Supplementary material for: Three-dimensional aromaticity in an antiaromatic cyclophane
Source: Nat Commun. 2019 Aug 8;10:3576. doi: 10.1038/s41467-019-11467-4 (PMC6687811; doi:10.1038/s41467-019-11467-4)
Supplement: Supplementary file 1 — Supplementary Information [file 41467_2019_11467_MOESM1_ESM.pdf]

Supplementary Information

Three-dimensional aromaticity in an antiaromatic cyclophane

Nozawa *et al*.

Supplementary Figures

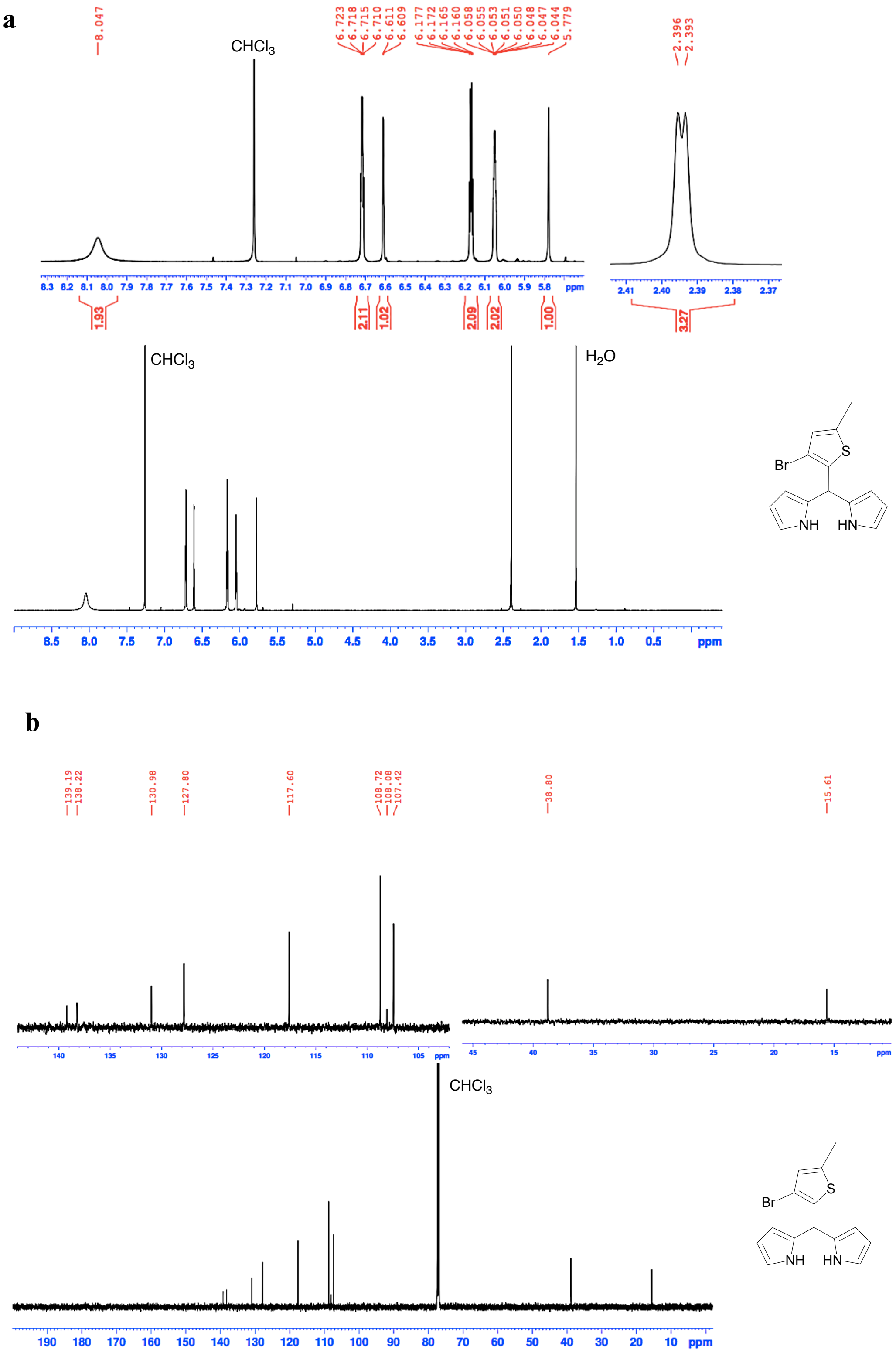

Supplementary Figure 1. NMR spectra of **7**. **a** ^{1}H NMR spectrum (500 MHz, $CDCl_3$). **b** ^{13}C NMR spectrum (126 MHz, $CDCl_3$).

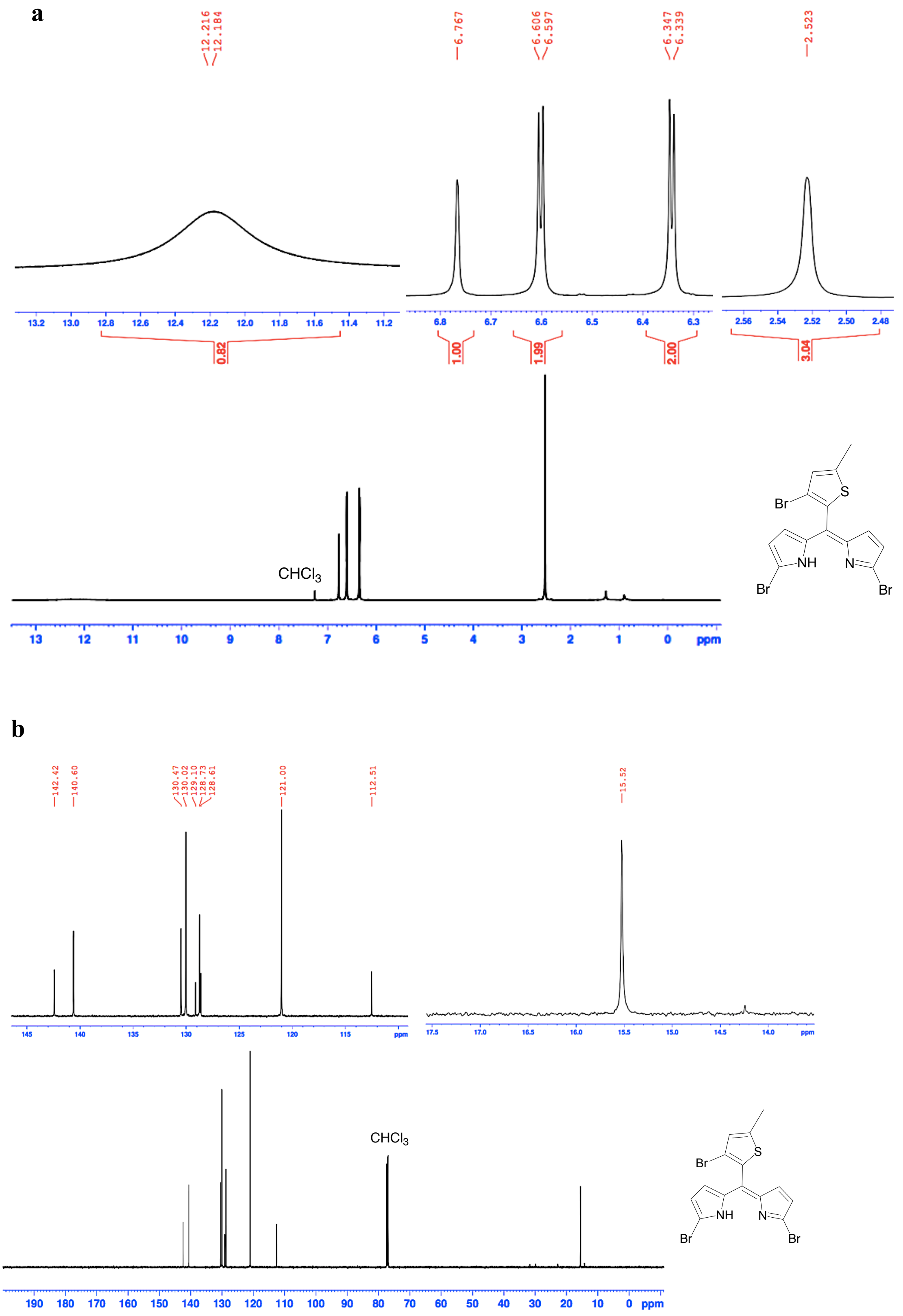

Supplementary Figure 2. NMR spectra of **8**. **a** ^{1}H NMR spectrum (500 MHz, $CDCl_3$). **b** ^{13}C NMR spectrum (126 MHz, $CDCl_3$).

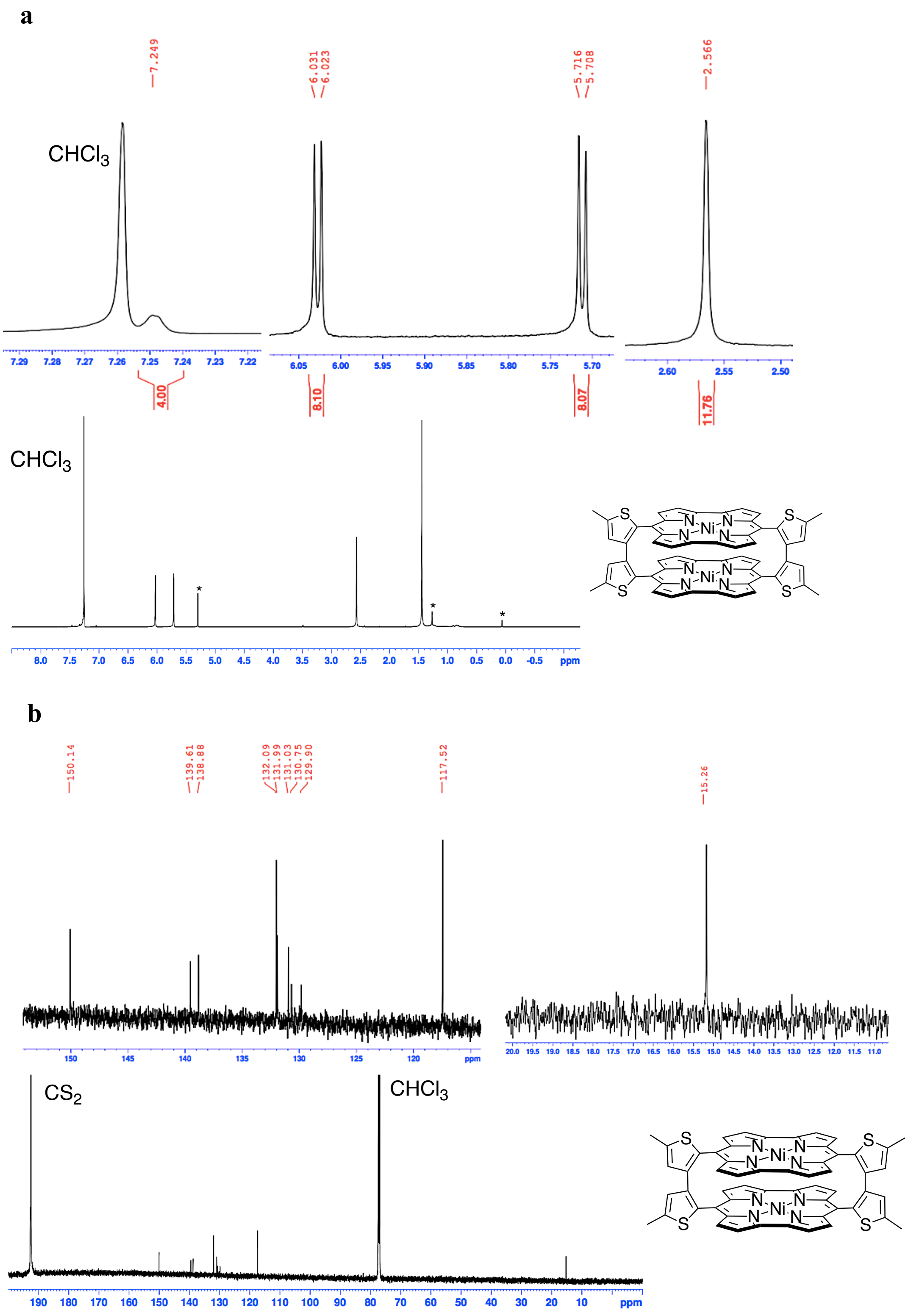

Supplementary Figure 3. NMR spectra of **5**. **a** ^{1}H NMR spectrum (500 MHz, $CDCl_3$). **b** ^{13}C NMR spectrum (126 MHz, $CDCl_3$).

a

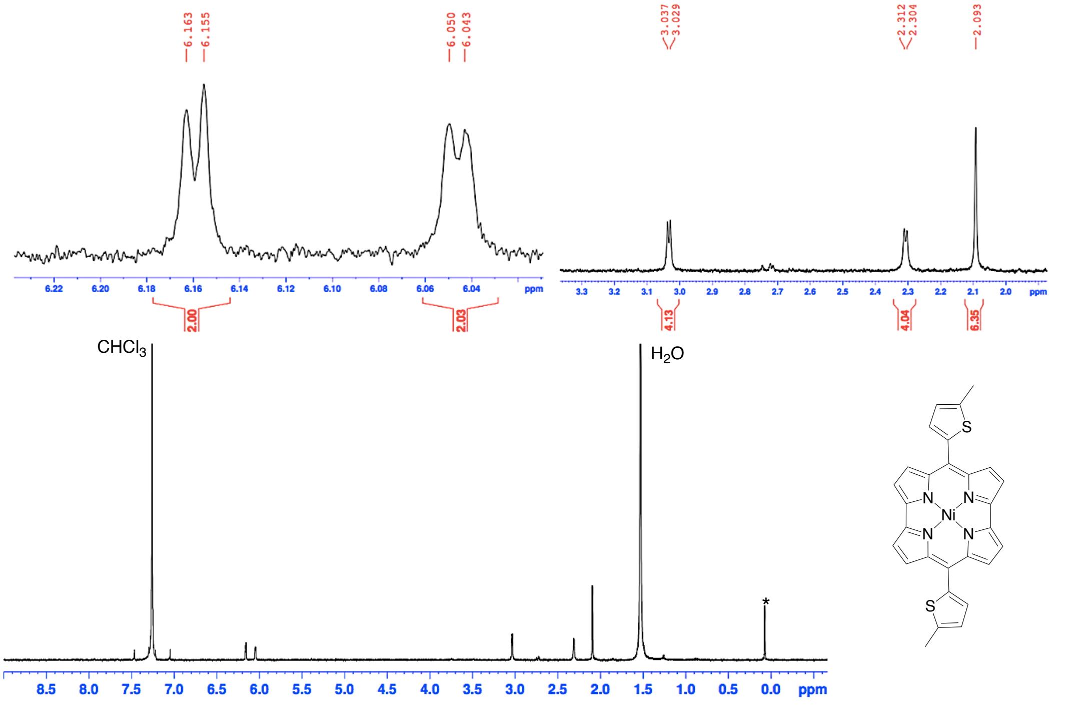

b

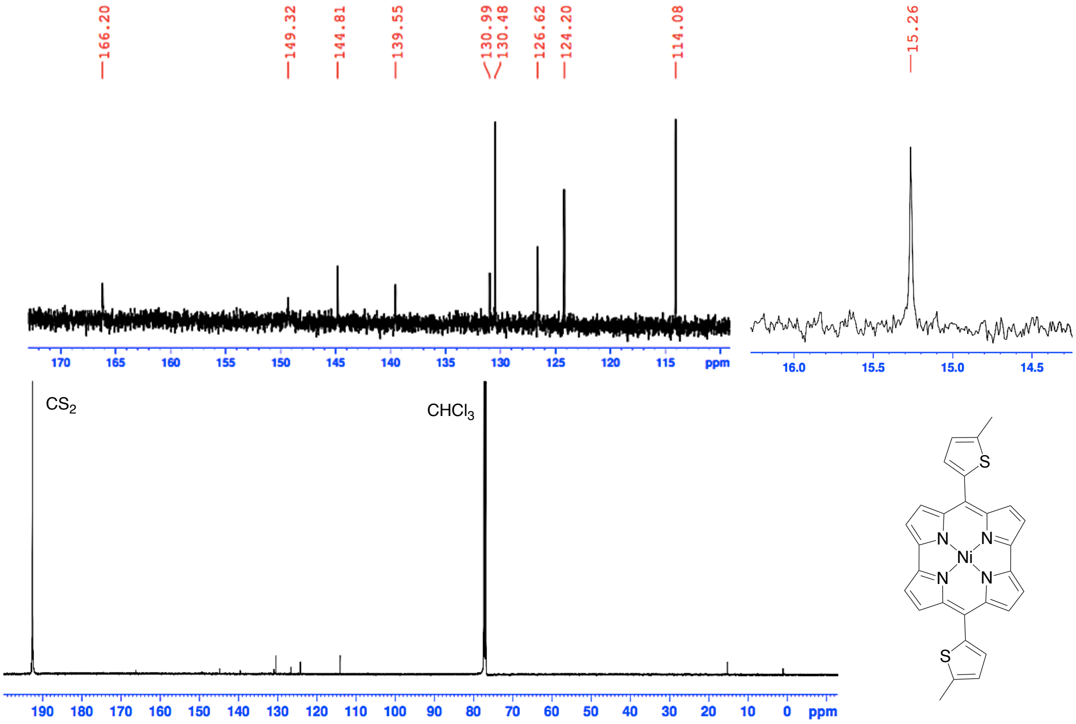

Supplementary Figure 4. NMR spectra of **6**. **a** ^{1}H NMR spectrum (500 MHz, $CDCl_3$). **b** ^{13}C NMR spectrum (126 MHz, $CDCl_3$).

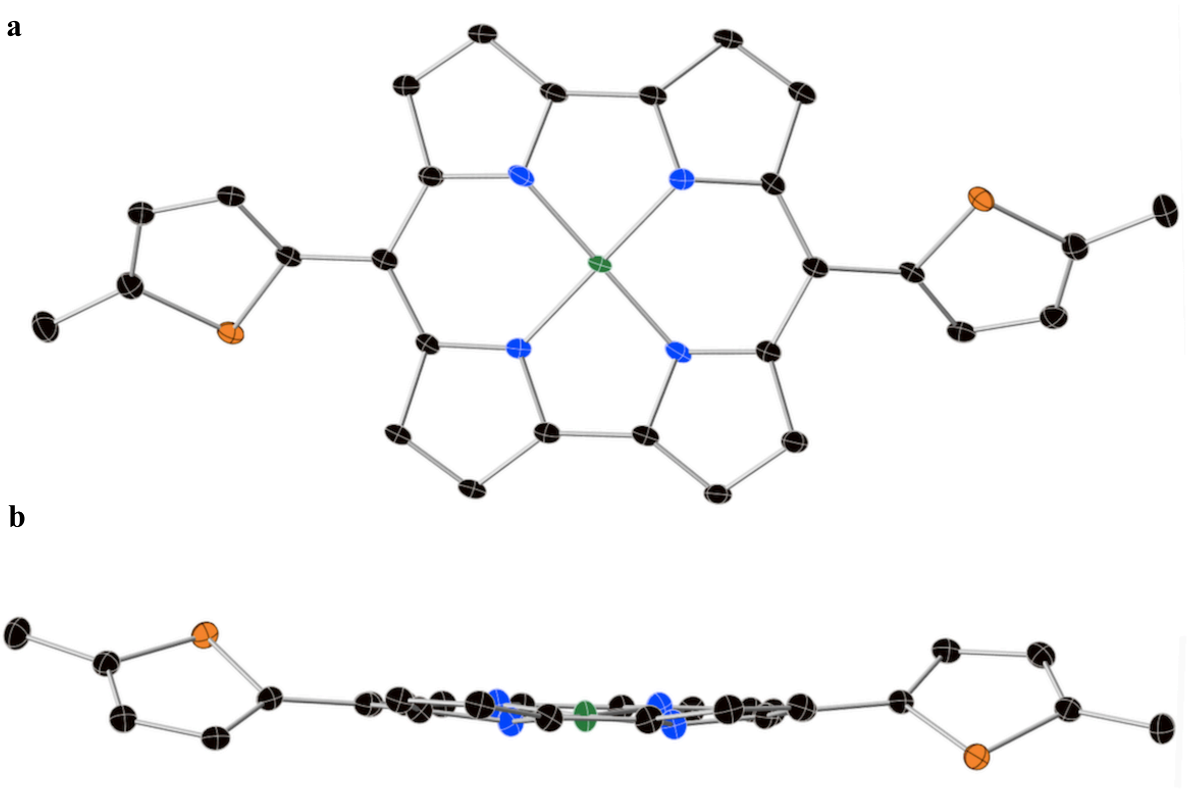

Supplementary Figure 5. X-ray crystal structure of **6**. **a** top view and **b** side view. Hydrogen atoms are omitted for clarity. The thermal ellipsoids are scaled at 50% probability level.

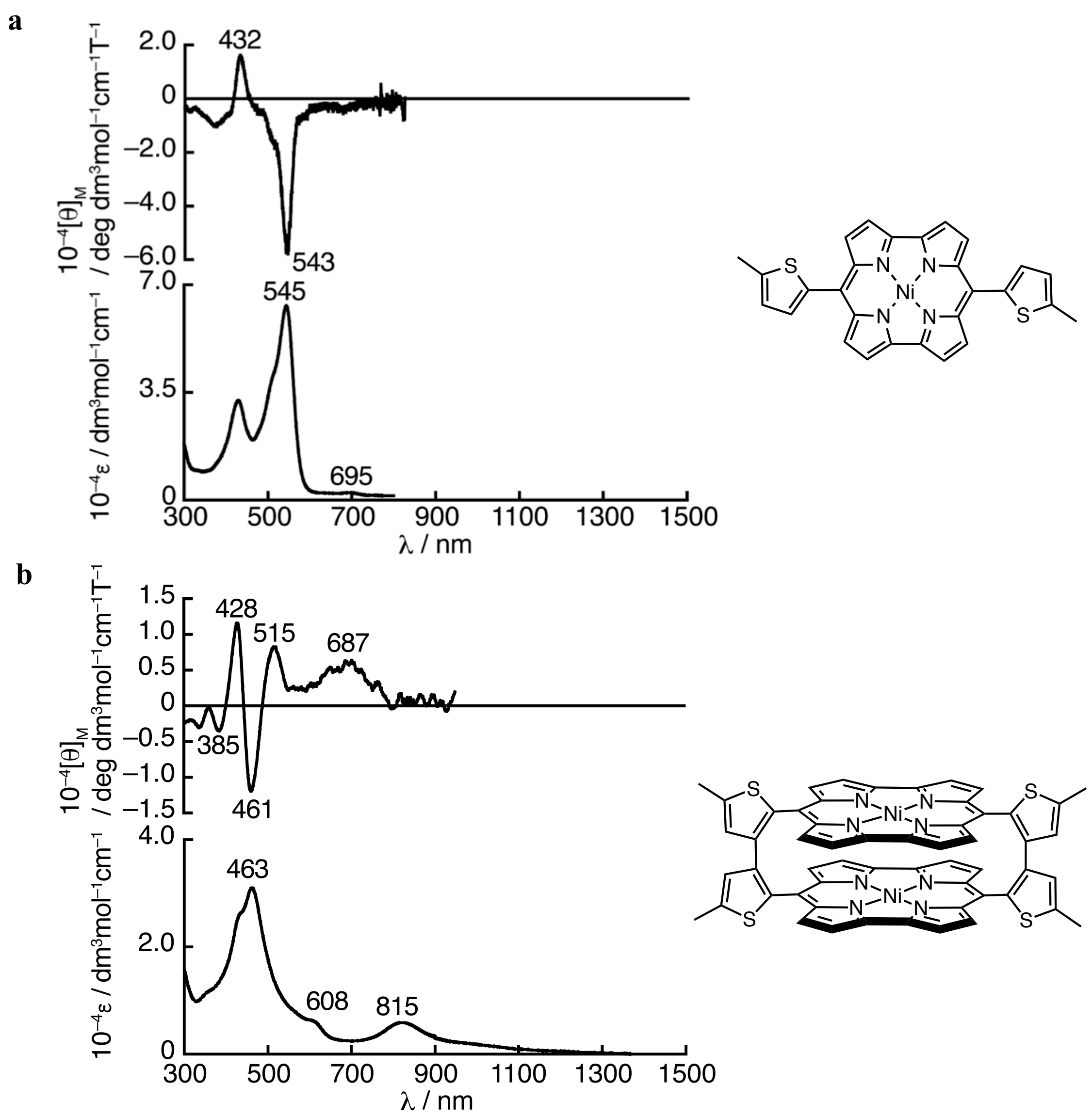

Supplementary Figure 6. **a** Absorption (bottom) and MCD (top) spectra of monomer **6** in CH_2Cl_2. **b** Absorption (bottom) and MCD (top) spectra of norcorrole cyclophane **5** in CH_2Cl_2.

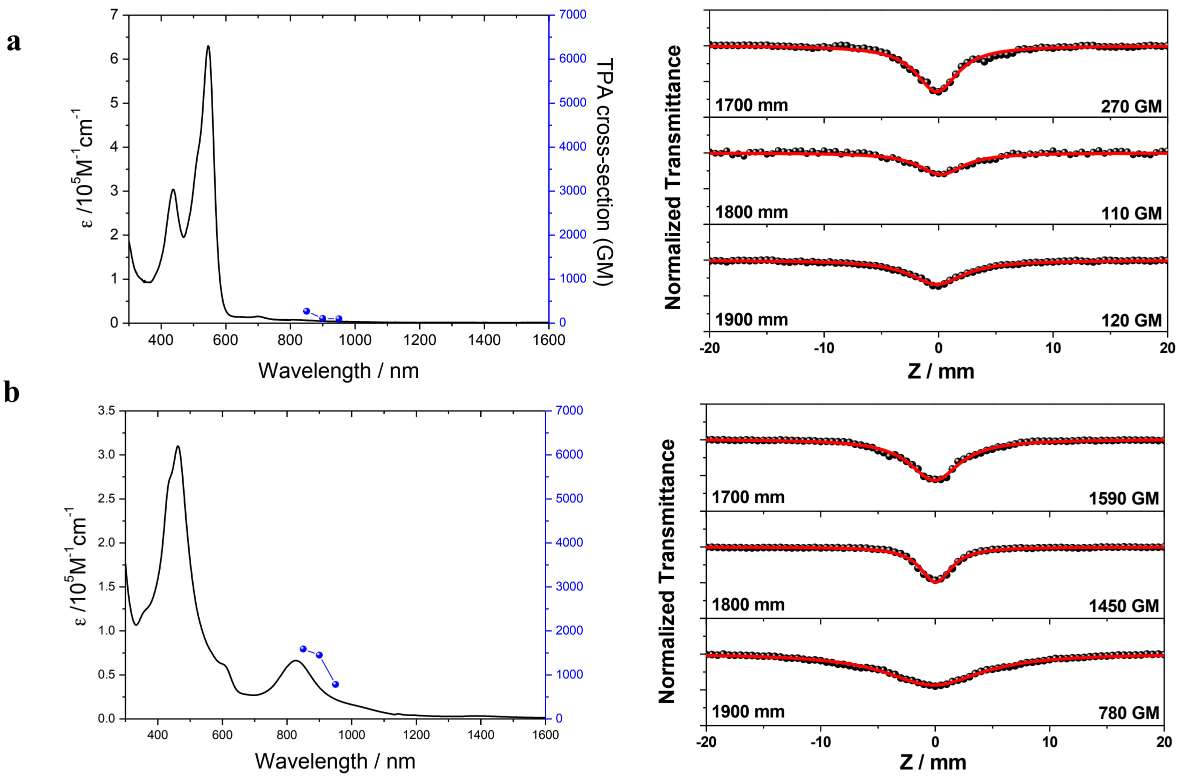

Supplementary Figure 7. **a** Two photon absorption cross-section values of **6** at 1700, 1800 and 1900 nm in 1,2-dichlorobenzene. **b** Two photon absorption cross-section values of **5** at 1700, 1800 and 1900 nm in 1,2-dichlorobenzene.

a

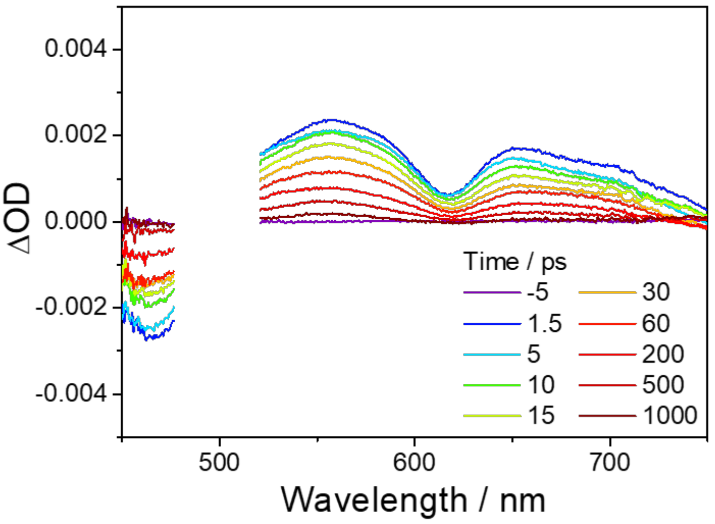

b

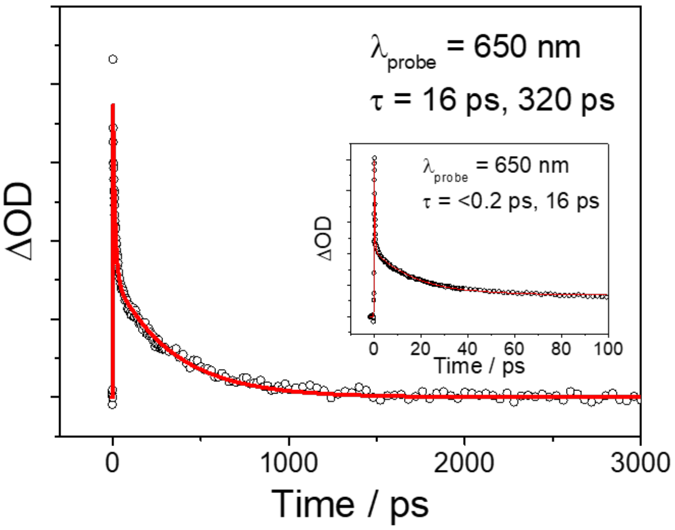

Supplementary Figure 8. **a** Transient absorption spectra of the cyclophane dimer **5** upon photoexcitation at 500 nm and **b** the decay profile. The inset shows the decay profile of short time window.

a

b

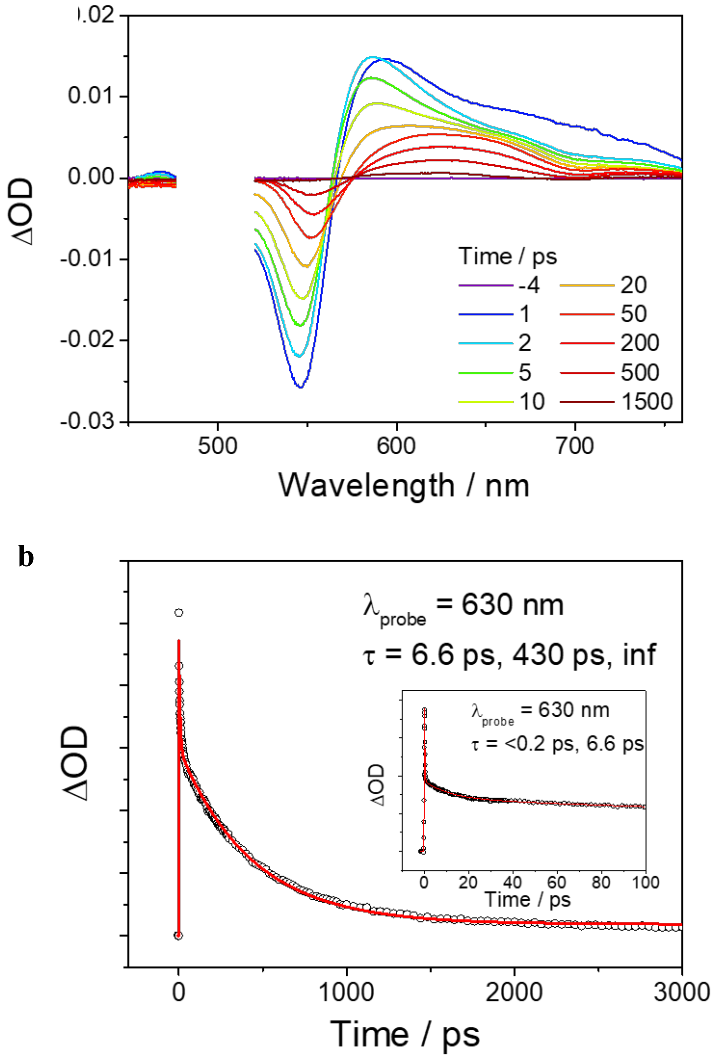

Supplementary Figure 9. **a** Transient absorption spectra of the monomer **6** and upon photoexcitation at 500 nm and **b** the decay profile. The inset shows the decay profile of short time window.

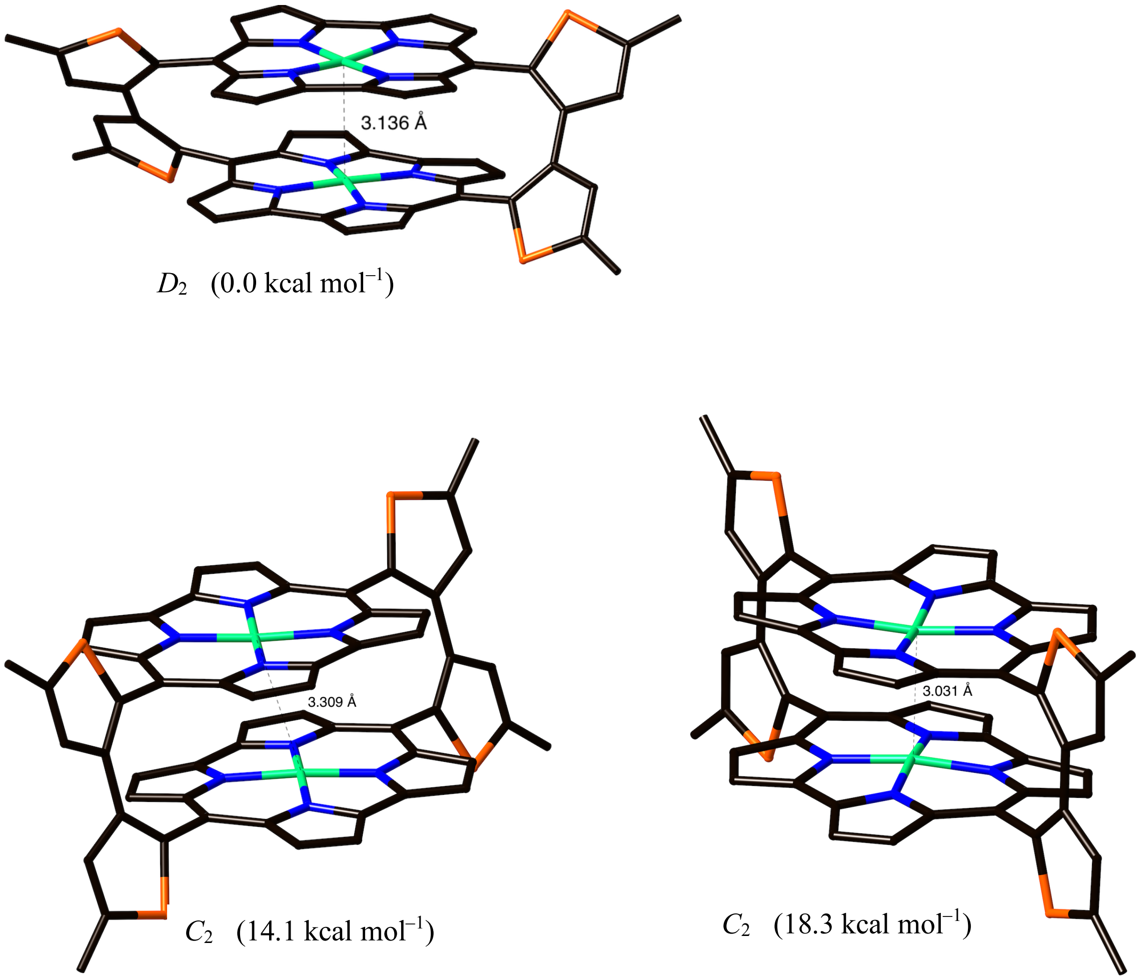

Supplementary Figure 10. Optimized structures of the cyclophane dimer **5** at the CAM-B3LYP/6-31G(d) level. The relative energy of each structure is shown in the parenthesis.

a

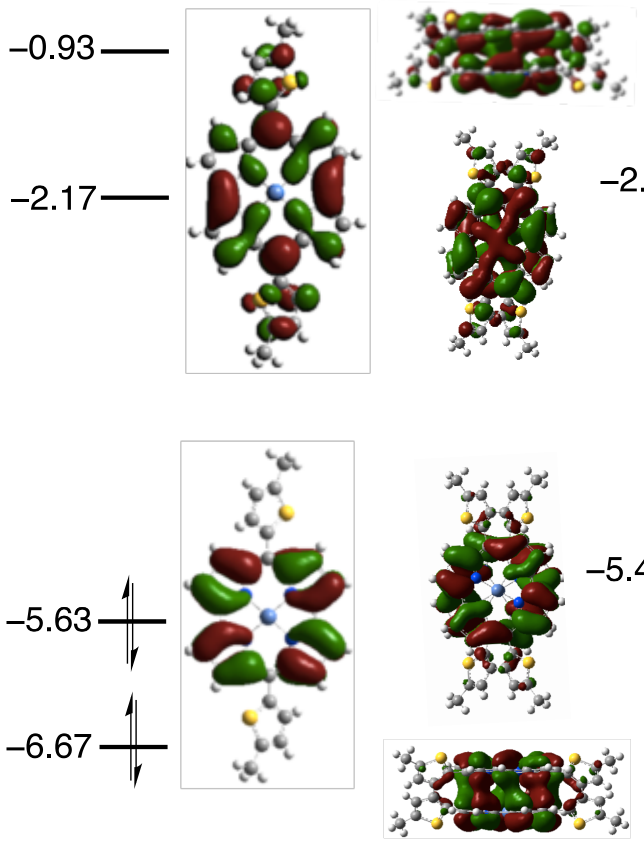

b

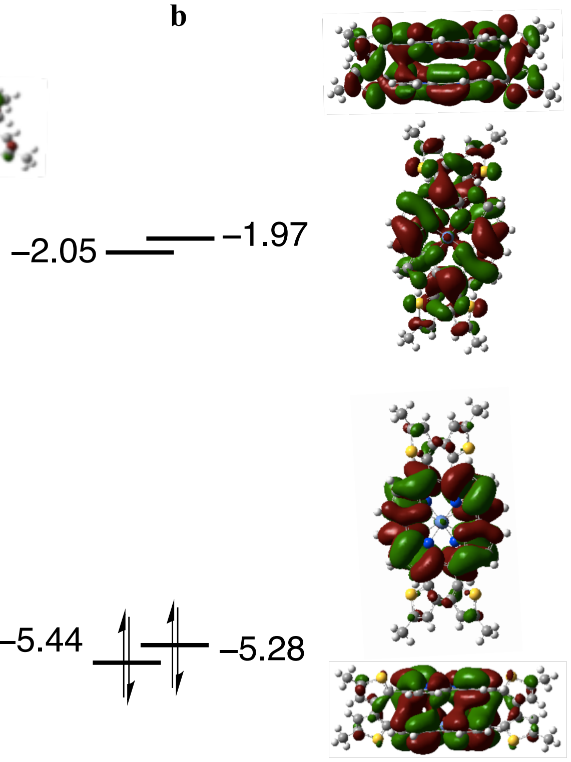

c

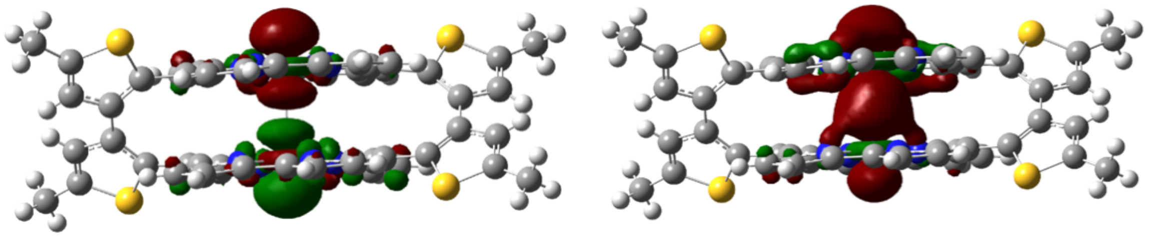

Supplementary Figure 11. **a** Frontier molecular orbitals and their energy levels (eV) of **6** at the CAM-B3LYP/6-31G(d). **b** Frontier molecular orbitals and their energy levels (eV) of **5** at the CAM-B3LYP/6-31G(d). **c** Interaction between two nickel atoms. Left: σ* (HOMO–4) and right: σ (HOMO–12).

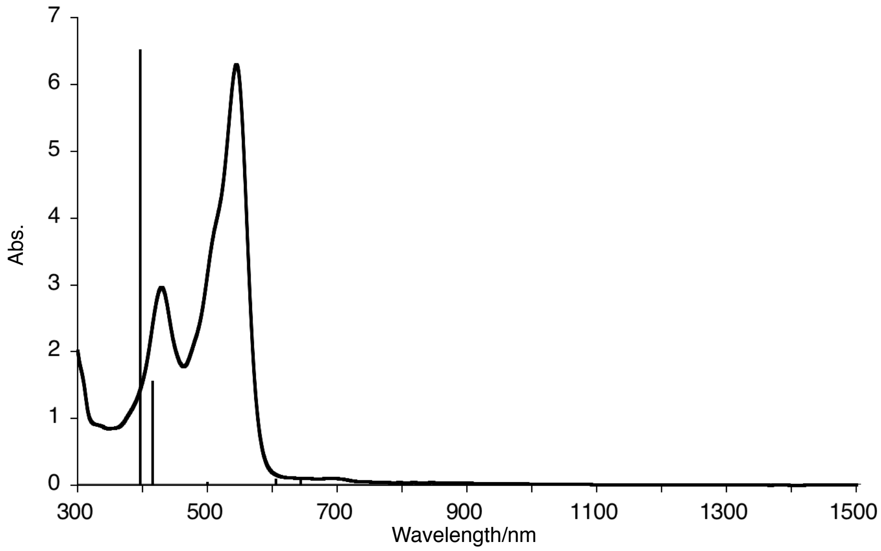

Supplementary Figure 12. Calculated absorption spectrum of **6**. The experimental spectrum of **6** is shown for comparison.

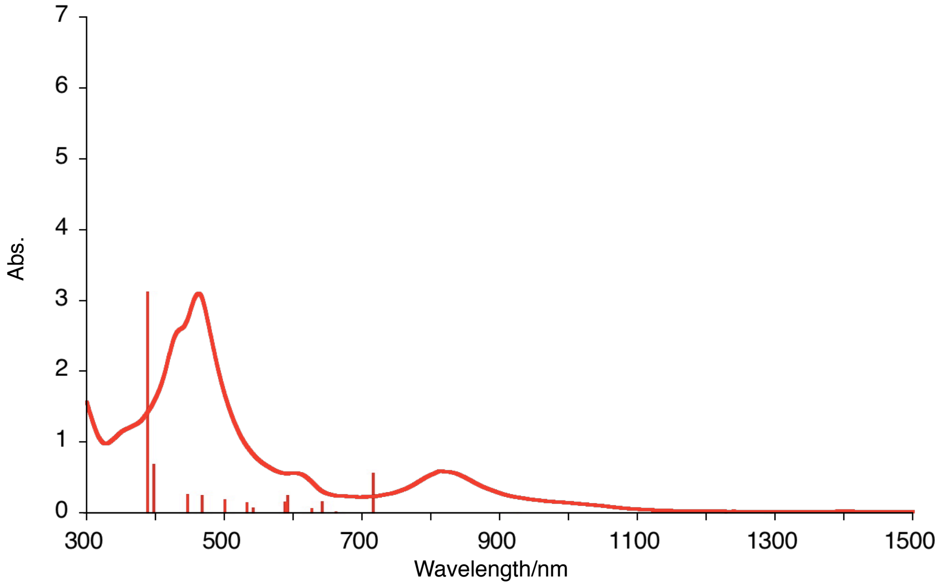

Supplementary Figure 13. Calculated absorption spectrum of **5**. The experimental spectrum of **5** is shown for comparison.

a

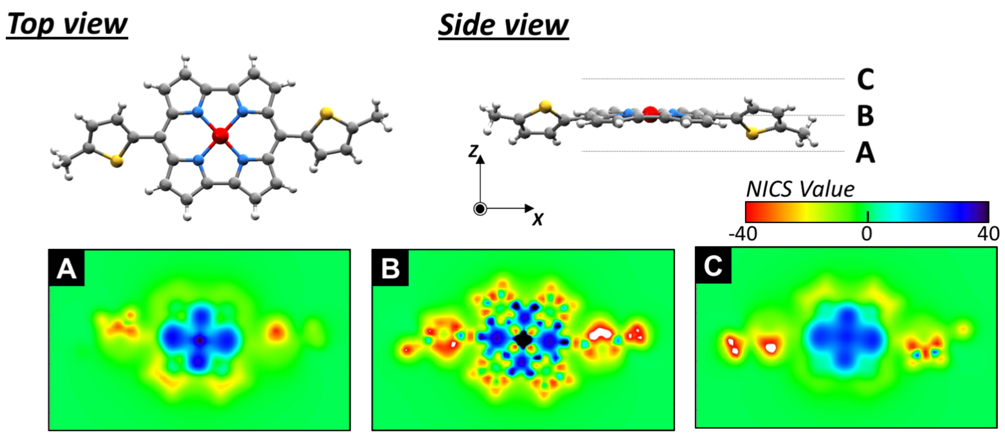

b

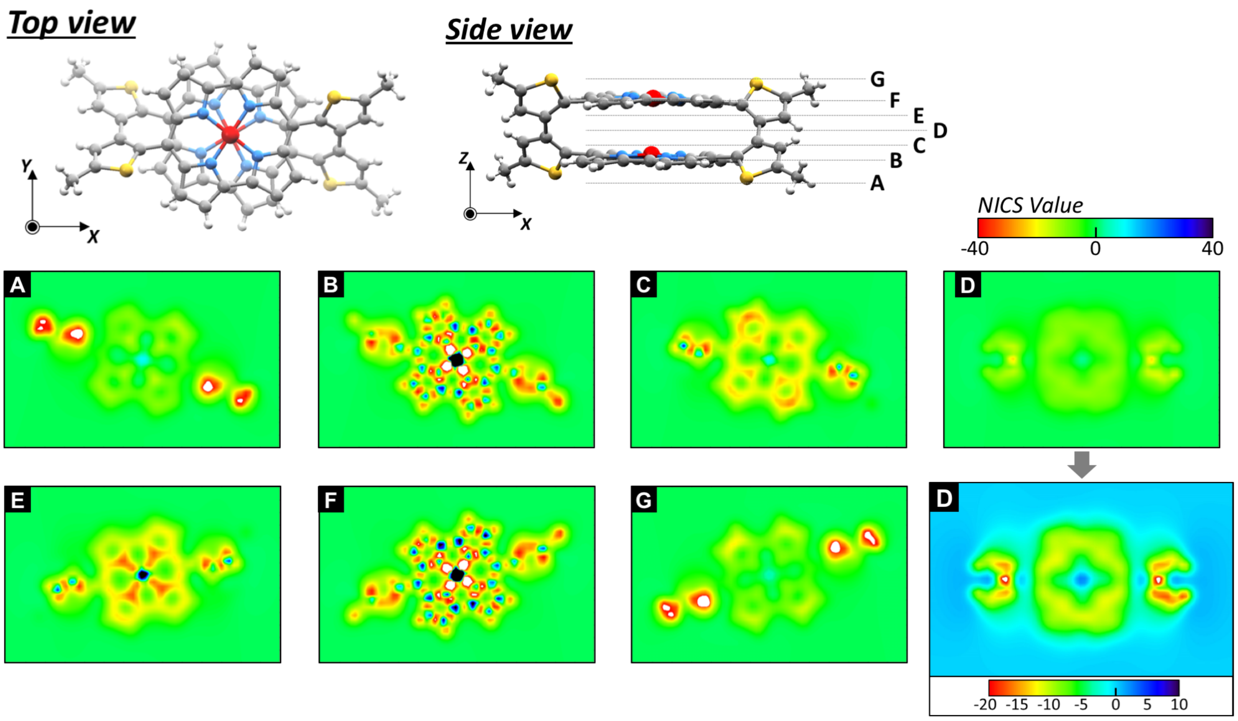

Supplementary Figure 14. **a** 2D-NICS plots of the magnetic shielding effect of **6** in various *xy*-planes. **b** 2D-NICS plots of the magnetic shielding effect of **5** in various *xy*-planes. Calculations were conducted on the X-ray structures at the GIAO-B3LYP/6-31G(d) level.

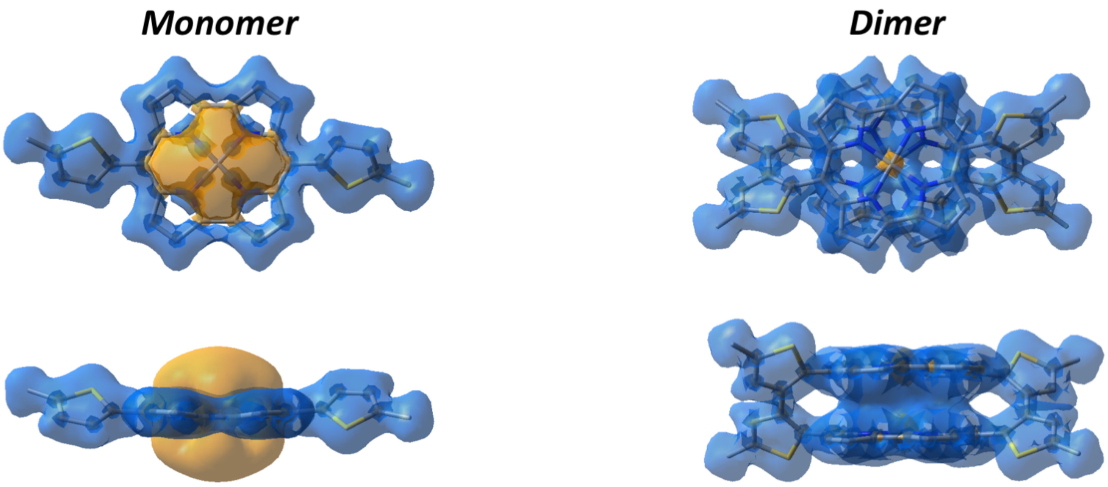

Supplementary Figure 15. 3D-isosurface plots of the magnetic shielding effect of **5** and **6**. The magnetic shielding effects are estimated based on the NICS values and represented blue (negative) and yellow (positive) colors.

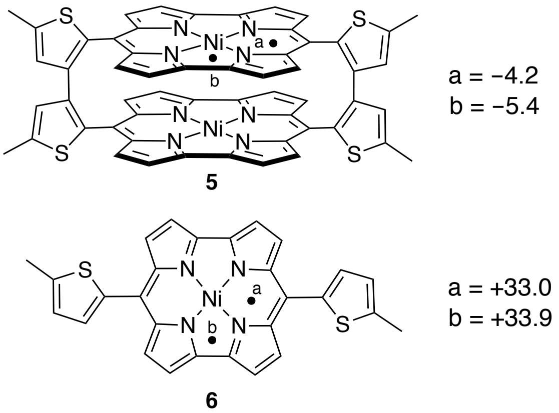

Supplementary Figure 16. NICS values of **5** and **6**.

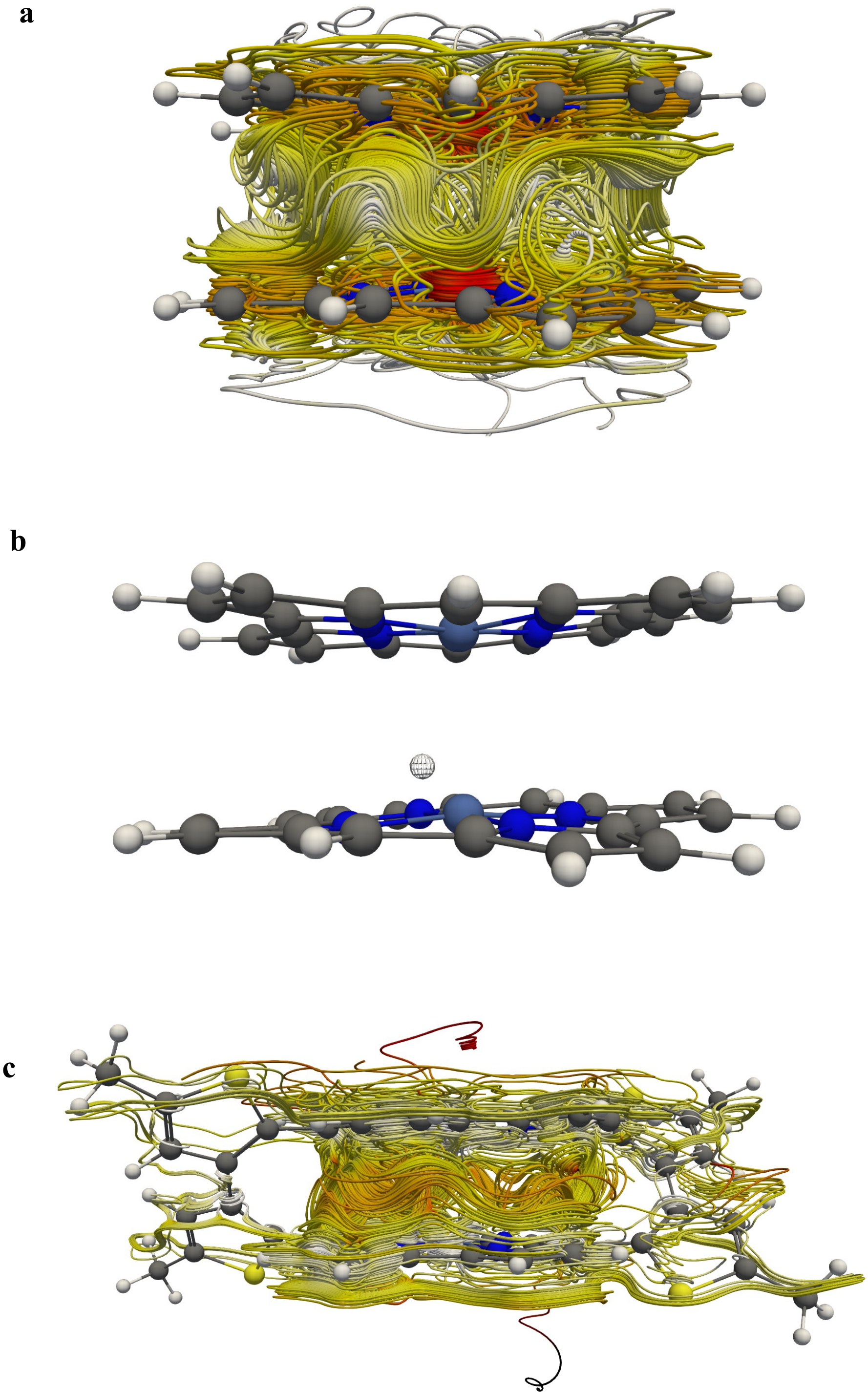

Supplementary Figure 17. **a** The calculated three-dimensional current density of **5’** represented as streamlines. **b** An inspection sphere of radius 0.3 bohr was placed above the carbon–carbon bond adjacent to the meso carbon atom. The calculation was performed on the model system **5’** without the bithiophene spacers. **c** The calculated three-dimensional current density of the real dimer **5** with the bithiophene spacers.

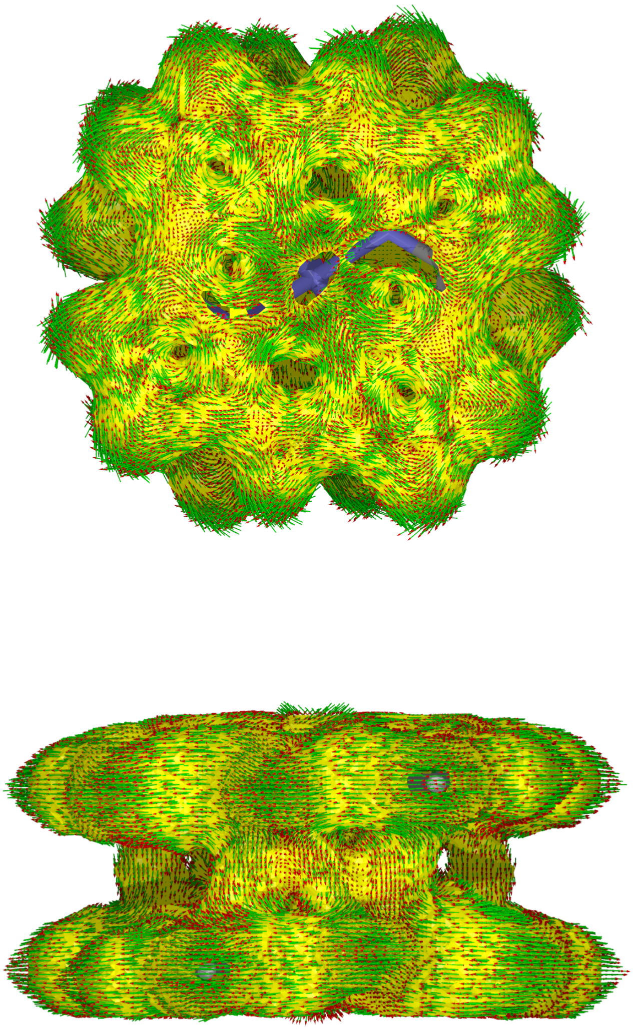

Supplementary Figure 18. Top and side views of ACID plots. The ACID calculation was conducted at the CSGT-B3LYP/6-31G(d) level on the model system **5’** without the bithiophene linkers.

Supplementary Tables

Supplementary Table 1. X-ray crystallographic data of **5** and **6**.

	**5**	**6**
empirical formula	$C_{62}H_{37}ClN_8Ni_2O_2S_4$	$C_{28}H_{18}N_4NiS_2$
formula weight	1191.10	533.30
habit	block	columnar
T, K	296(2)	296(2)
crystal system	Monoclinic	Monoclinic
space group	$P2_1/n$	$P2_1/n$
a, Å	13.87770(10)	13.69930(1)
b, Å	14.39280(10)	6.9517(1)
c, Å	26.5059(3)	23.2868(2)
α, deg	90.00	90.0000
β, deg	97.8426(4)	105.1879(3)
γ, deg	90.00	90.0000
V, Å^3	5244.74(8)	2140.22(2)
Z	4	4
Dc, g/cm^3	1.508	1.655
F(000)	2440	1096.00
crystal size, mm^3	0.07 × 0.05 × 0.03	0.10 × 0.05 × 0.03
$2\theta_{max}$, °	65.00	65.00
R_{int}	0.059	0.047
R_1 ($I > 2\sigma(I)$)	0.0866	0.0450
wR_2 (all data)	0.2398	0.1266
GOF	1.061	1.071
observed reflections	10306	4312
No. of unique reflections	10575	4359
No. of reflections measured	10575	4359
parameters	781	316

Supplementary Table 2. Selected oscillator strengths and wavelengths for **6**.

compound	wavelength (nm)	oscillator strength	component	population
**6**	1194.96	0.0000	132 → 133	0.69944
	642.12	0.0095	129 → 133	0.17484
			131 → 133	0.66634
	603.73	0.0100	129 → 133	–0.10175
			130 → 133	0.66828
			132 → 134	0.11262
	499.64	0.0012	128 →133	0.69233
	414.85	0.2609	130 → 133	–0.12063
			132 → 134	0.68286
	412.76	0.0041	126 → 133	–0.15086
			127 → 133	0.58093
			130 → 134	0.31501
	396.39	1.1065	126 → 134	–0.19320
			129 → 133	0.63009
			131 → 133	–0.18388

Supplementary Table 3. Selected oscillator strengths and wavelengths for **5**.

compound	wavelength (nm)	oscillator strength	component	population
**5**	1297.53	0.0005	261 → 263	–0.40670
			262 → 263	0.33630
			262 → 264	0.45568
	1230.90	0.0024	261 → 263	0.20126
			261 → 264	–0.39062
			262 → 263	0.48515
			262 → 264	–0.24948
	928.10	0.0001	261 → 263	0.52969
			262 → 264	0.46206
	720.37	0.0836	257 → 263	–0.19041
			261 → 264	0.55253
			262 → 263	0.35304
	643.63	0.0231	252→ 263	–0.16467
			255 → 263	–0.12313
			256 → 264	0.10171
			259 → 263	0.39164
			259 → 264	–0.26275
			260 → 263	–0.19819
			260 → 264	–0.39105
	595.71	0.0359	244 → 264	-0.10793
			257 → 263	0.64219
			261 → 264	0.13354
			262 → 263	0.12453
	561.89	0.0224	258 → 580	0.25211
			259 → 580	–0.20162
			262 → 581	0.58402
	424.48	0.0157	248 → 264	0.12614
			251 → 263	0.36497
			254 → 263	0.29770
			254 → 264	0.19445
			255 → 263	0.11097
			256 → 265	–0.13097
			259 → 265	–0.17858
			260 → 265	0.27106

393.64	0.4675	252 → 263	–0.30674
		252 → 264	0.20917
		253 → 264	0.34856
		255 → 263	–0.11529
		256 → 264	0.15542
		258 → 263	–0.12915
		259 → 263	–0.11238
		261 → 266	–0.33187

Supplementary Methods

Materials and characterization. ^{1}H NMR (500 MHz) and ^{13}C NMR (126 MHz) spectra were recorded on a Bruker AVANCE III HD spectrometer. Chemical shifts were reported as the delta scale in ppm relative to $CHCl_3$ (δ = 7.260 ppm) for ^{1}H NMR and $CDCl_3$ (δ = 77.16 ppm) for ^{13}C NMR. UV/vis/NIR absorption spectra were recorded on a Shimadzu UV-2550 or JASCO V670 spectrometer. Magnetic circular dichroism (MCD) spectra were recorded on a JASCO J-1500 spectrodichrometer by applying parallel and antiparallel magnetic fields to the light propagation with a 1.6 T permanent magnet (T = tesla). High-resolution mass spectra were recorded on a Bruker microTOF using positive mode ESI-TOF method for acetonitrile solutions. Unless otherwise noted, materials obtained from commercial suppliers were used without further purification.

Synthesis of compounds. *meso*-5-Methyl-3-bromo-2-thienyl-dipyrromethane **7**. To a mixture of 5-methyl-3-bromo-2-thiophenealdehyde (2.05 g, 10.0 mmol) and pyrrole (20 mL), trifluoroacetic acid (300 μL, 3.92 mmol) was added. The mixture was stirred at room temperature for 2 h. The reaction mixture was extracted with ethyl acetate. The organic layer was washed with water, dried over anhydrous Na_2SO_4 and concentrated in vacuo. Purification by silica-gel column chromatography with CH_2Cl_2/hexane afforded **7** in 36% (1.17 g, 3.64 mmol) as a white solid. ^{1}H NMR (500 MHz, $CDCl_3$): δ = 8.05 (s, 2H), 6.72 (dd, J = 4.0, 2.5 Hz, 2H), 6.61 (d, J = 1.0 Hz, 1H), 6.17 (dd, J = 5.0, 2.5 Hz, 2H), 6.06–6.04 (m, 2H), 5.78 (s, 1H), 2.39 (d, J = 1.5 Hz, 3H) ppm; ^{13}C NMR (126 MHz, $CDCl_3$): δ = 139.2, 138.2, 131.0, 127.8, 117.6, 108.7, 108.1, 107.4, 38.8, 15.6 ppm; ESI-MS: m/z = 321.0025, calcd for $(C_{14}H_{13}BrN_2S)^+$ = 321.0056 $[(M+H)^+]$.

meso-5-Methyl-3-bromo-2-thienyl-α,α'-dibromodipyrrin **8**. A solution of **7** (1.16 g, 3.61 mmol) in dry THF (75 mL) was cooled at –78 °C and *N*-bromosuccinimide (1.15 g, 6.50 mmol) was added in four potions at 10 min interval. After stirring for 1 h, 2,3-dichloro-5,6-dicyano-1,4-benzoquinone (1.14 g, 5.02 mmol) was added. The resulting mixture was stirred at –78 °C for 10 min then warmed to room temperature. After stirring for additional 30 min, the reaction mixture was filtered through a short pad of alumina column (EtOAc as an eluent) and then evaporated. Purification by silica-gel column chromatography with CH_2Cl_2/hexane afforded **8** in 98% (1.52 g, 3.19 mmol) as an orange solid. ^{1}H NMR (500 MHz, $CDCl_3$): δ = 12.20 (br, 1H), 6.77 (s, 1H), 6.61 (d, J = 4.5 Hz, 2H), 6.34 (d, J = 4.0 Hz, 2H), 2.52 (s, 3H) ppm; ^{13}C NMR (126 MHz, $CDCl_3$): δ = 142.4, 140.6, 130.5, 130.0, 129.1, 128.7, 128.6, 121.0, 112.5, 15.5 ppm; ESI-MS: *m/z* = 478.8052, calcd for $(C_{14}H_9Br_3N_2S)^+$ = 478.8068 $[(M+H)^+]$.

meso-5-Methyl-3-bromo-2-thienyl-α,α'-dibromodipyrrin Ni(II) complex **3**. To a solution of **8** (1.52 g, 3.19 mmol) in CH_2Cl_2 (30 mL) and methanol (15 mL), $Ni(OAc)_2$•$4H_2O$ (396 mg, 1.60 mmol) was added. After 1 h of stirring at room temperature, the mixture was evaporated under reduced pressure. The solid residue was recrystallized from CH_2Cl_2/MeOH to afford **3** as a brown solid (1.20 g, 1.18 mmol, 75%). HR-MS (ESI-MS): *m/z* = 1004.5365, calcd for $(C_{30}H_{18}Br_4N_4Ni)^+$ = 1004.5343 $[(M+H)^+]$.

Norcorrole cyclophane **5**. To a mixture of **3** (51.8 mg, 51.3 µmol), $Ni(cod)_2$ (69.3 mg, 125 µmol) and 2,2'-bipyridine (40.2 mg, 125 µmol), dry THF (20 mL) was added. The solution was stirred at 90 °C for 12 h. The mixture was through alumina pad, and evaporated under reduced pressure to solid residue. Purification by silica-gel column chromatography with $CHCl_3$/hexane and recrystallization from CH_2Cl_2/MeOH afforded **5** in 7% (1.99 mg, 1.87 µmol) as a black solid and a small amount of **6** as a byproduct as a brown solid. *meso*-Di(4-bromo-5-methyl-2-thienyl)norcorrole Ni(II) **4** was not stable and rapidly decomposed under ambient conditions. The decomposition products were not eluted from the silica-gel column. Norcorrole cyclophane **5**: ^{1}H NMR (500 MHz, $CDCl_3$): δ = 7.25 (s, 4H), 6.03 (d, J = 4.0 Hz, 8H), 5.71 (d, J = 4.0 Hz, 8H), 2.57 (s, 12H) ppm; ^{13}C NMR (126 MHz, $CDCl_3$): δ = 150.1, 139.6, 138.9, 132.1, 132.0, 131.0, 130.8, 129.9, 117.5, 15.3 ppm; UV-Vis-NIR (CH_2Cl_2): λ_{max} (ε [M^{-1} cm^{-1}]) 463 (31000), 608 (6000), 815 (6000) nm; ESI-MS: *m/z* = 1060.0251, calcd for $(C_{56}H_{32}N_8Ni_2S_4)^+$ = 1060.0334 $[M^+]$. *meso*-Di(5-methyl-2-thienyl)norcorrole Ni(II) **6**. ^{1}H NMR (500 MHz, $CDCl_3$): δ = 6.16 (d, J = 4.0 Hz, 2H), 6.05 (d, J = 3.5 Hz, 2H), 3.03 (d, J = 4.0 Hz, 4H), 2.31 (d, J = 4.0 Hz, 4H), 2.09 (s, 6H) ppm; ^{13}C NMR (126 MHz, $CDCl_3$): δ = 166.2, 149.3, 144.8, 139.6, 131.0, 130.5, 126.6, 124.2, 114.1,

15.3 ppm; UV-Vis-NIR (CH_2Cl_2): λ_{max} (ε [M^{-1} cm^{-1}]) 545 (63000), 695 (1000) nm; ESI-MS: m/z = 532.0347, calcd for $(C_{28}H_{18}N_4NiS_2)^+$ = 532.0321 [M^+].

X-ray Diffraction Analysis. X-ray diffraction data of **5** and **6** were collected on CCD (MX225HE, Rayonix) with the synchrotron radiation (λ = 0.8000 Å) monochromated by the fixed exit Si (111) double crystal at the BL38B1 in the SPring-8 with approval of the Japan Synchrotron Radiation Research Institute (JASRI) (proposal Nos. 2015B1397, 2016A1121). The oscillation angle, camera distance, and exposure time per frame were 1°, 75 mm, and 1s, respectively. Two data sets consisted of 180 frames were integrated, scaled, and merged with the programs HKL2000[1]. The structure was solved by SHELXT[2] and refined by least-squares calculations (SHELXL)[3] on F^2 for all reflections using the crystallographic software packages CrystalStructure[4]. All non-hydrogen atoms were refined with anisotropic displacement parameters and hydrogen atoms were placed in idealized positions and refined as rigid atoms with the relative isotropic displacement parameters. The detailed crystallographic data for both compounds is listed in Supplementary Table 1.

HOMA calculations. HOMA values of **5** and **6** were calculated using C–C and C–N bond lengths of the X-ray crystallographic structures, according to the following equations:

$$\mathrm{HOMA} = 1 - \alpha/n \sum \left(R_{\mathrm{opt}} - R_{\mathrm{i}}\right)^2 \quad (1)$$

where n is the number of bonds taken into summation, α is an empirical constant, R_{opt} is an optimal bond length, and R_i is a bond length of i th bond. R_{opt} = 1.388 (C–C) and 1.334 (C–N) Å and α = 257.7 (C–C) and 93.52 (C–N) were used.

Two Photon Absorption Measurement. The two-photon absorption spectrum was measured in the NIR region using the open-aperture Z-scan method with 176 fs pulses from an optical parametric amplifier (Light Conversion, ORPHEUS) operating at a repetition rate of 10 kHz generated from a Yb:KGW regenerative amplifier system (Light Conversion, PHAROS). After passing through a 10 cm focal length lens, the laser beam was focused and passed through a 1 mm quartz cell. Since the position of the sample cell could be controlled along the laser beam direction (z axis) using the motored controlled delay stage, the local power density within the sample cell could be simply controlled under constant laser intensity.

The transmitted laser beam from the sample cell was then detected by the same photodiode as used for reference monitoring. The on-axis peak intensity of the incident pulses at the focal point, I_0, ranged from 60 to 130 GW cm^{-2}. For a Gaussian beam profile, the nonlinear absorption coefficient can be obtained by curve fitting of the observed open-aperture traces $T(z)$ with the following equation:

$$T(z) = 1 - \frac{\beta I_0(1-e^{-\alpha_0 l})}{2\alpha_0(1+(z/z_0)^2)} \tag{2}$$

where α_0 is the linear absorption coefficient, l is the sample length, and z_0 is the diffraction length of the incident beam. After the nonlinear absorption coefficient has been obtained, the TPA cross-section σ_2 of one solute molecule (in units of GM, where 1 GM = 10^{-50} cm^4 s photon^{-1} molecule^{-1}) can be determined by using the following relationship:

$$\beta = \frac{\sigma_2 N_A C}{h\nu} \tag{3}$$

where N_A is the Avogadro constant, C is the concentration of the compound in solution, h is the Planck constant, and v is the frequency of the incident laser beam.

Femtosecond Transition Absorption Measurements. The femtosecond time-resolved transient absorption (fs-TA) spectrometer consisted of an optical detection system and an optical parametric amplifier (OPA; Palitra, Quantronix) pumped by a Ti:sapphire regenerative amplifier system (Integra-C, Quantronix) operating at a 1 kHz repetition rate. The generated OPA pulses, which were used as pump pulses, had a pulsewidth of ~150 fs and an average power of 100 mW in the range of 280–2700 nm. White light continuum (WLC) probe pulses were generated using a sapphire window (4 mm thick) by focusing a small portion of the fundamental 800 nm pulses, which was picked off by a quartz plate before entering the OPA. The time delay between the pump and probe beams was carefully controlled by making the pump beam travel along a variable optical delay (ILS250, Newport). The intensities of the spectrally dispersed WLC probe pulses were monitored by a high speed spectrometer (Ultrafast Systems) for both visible and near-infrared measurements. To obtain the time-resolved transient absorption difference signal (ΔA) at a specific time, the pump pulses were chopped at 500 Hz and absorption spectra intensities were saved alternately with or without pump pulses. Typically, 4000 pulses excited the samples to obtain the fs-TA

spectra at each delay time. The polarization angle between the pump and probe beam was set at the magic angle (54.7°) using a Glan-laser polarizer with a half-wave retarder to prevent polarization-dependent signals. Cross-correlation fwhm in the pump-probe experiments was less than 200 fs. After the fs-TA experiments, the absorption spectra of all compounds were carefully examined to determine if there were artifacts due to degradation and photo-oxidation of samples.

Molecular Orbital and NICS Calculations. Calculations were carried out using the *Gaussian 09* program[5]. Geometries of **5** and **6** for NICS calculations were obtained from their X-ray structures. Molecular orbital calculations were performed with the CAM-B3LYP functional[6] and the 6-31G(d) basis set was used for all atoms. The NICS calculations were performed with by the DFT method at the GIAO methods at the B3LYP/6-31G(d) level. The calculated NICS values were analyzed with Multiwfn 3.3.9 program, producing the two-dimensional maps and three-dimensional isosufaces[7]. The Wiberg bond index was obtained with NBO 5.0, which is implemented in the *Gaussian 09* program.

Energy Decomposition Analysis. We choose to employ the adiabatic absolutely localized molecular orbital EDA (adiabatic ALMO-EDA) of Head-Gordon and co-workers[8]. The adiabatic ALMO-EDA calculations were performed at the ωB97XD[9]/6-31G(d) level in Q-Chem 5.0[10]. The geometry of the stacked norcorrole dimer was taken from the X-ray structure of **5** but the bithiophene linkers were replaced by hydrogen atoms to obtain the model system **5'**. For the EDA analysis of the corresponding free-base norcorrole dimer, each nickel atom was replaced with two hydrogen atoms.

GIMIC Calculation. The molecular structure of **5** was optimized at the TPSSh[11] DFT level using Turbomole 7.2[12]. We employed the Karlsruhe def2-TZVP basis sets[13]. Dispersion effects have been considered by using the Becke-Johnson damped D3 correction[14]. For GIMIC analysis, we employed a model system **5'**, in which the bithiophene linkers were replaced by hydrogen atoms, as well as the real dimer **5**. The molecule was oriented in the xy plane such that the Ni–Ni axis served as z-axis. The external magnetic field was applied along the z-axis. Nuclear magnetic shieldings were calculated using the B3LYP functional as implemented in Turbomole[15]. Magnetically induced current densities were calculated using the GIMIC program[16-18]. The density matrices were obtained from nuclear magnetic shielding calculations on B3LYP/def2-TZVP level of theory.

The visualization of the three-dimensional current density has been done with the streamline plugin of ParaView 5.6[19] using an inspection sphere of radius 0.3 bohr. The inspection sphere has been placed between the meso carbon atom and an adjacent carbon atom as shown in Supplementary Fig. S17b. The three-dimensional animated current streamlines are obtained with ParaView. For the design of the schematic visualization of the three-dimensional current pattern of **5** VMD[20] and Powerpoint have been employed.

Supplementary References

(1) Otwinowski, Z.; Minor, W. Processing of X-ray diffraction data collected in oscillation mode. *Methods Enzymol*. **276**, 307-326 (1997).

(2) Sheldrick, G. M. SHELXT – Integrated space-group and crystal-structure determination. *Act. Crystallogra., Sect. A,* **71**, 3-8 (2015).

(3) Sheldrick, G. M. A short history of SHELX. *Acta Crystallogra., Sect. A,* 64, 112-122 (2008).

(4) *CrystalStructure Version 4.2*. Rigaku Corporation, Tokyo, Japan.

(5) Frisch, M. J. *et al. Gaussian 09, Revision D.01* (Gaussian, Inc., Wallingford CT, 2013).

(6) Yanai, T., Tew, D. & Handy, N. A new hybrid exchange–correlation functional using the Coulomb-attenuating method (CAM-B3LYP). *Chem. Phys. Lett.* **393**, 51-57 (2004).

(7) Lu, T. & Chen, F. Multiwfn: a multifunctional wavefunction analyzer. *J. Comput. Chem*. **33**, 580 (2012).

(8) Mao, Y., Horn, P. R. & Head-Gordon, M. Energy decomposition analysis in an adiabatic picture. *Phys. Chem. Chem. Phys.* **19**, 5944-5958 (2017).

(9) Chai, J.-D. & Head-Gordon, M. Long-range corrected hybrid density functionals with damped atom–atom dispersion corrections. *Phys. Chem. Chem. Phys.* **10**, 6615-6620 (2008).

(10) Shao, Y. *et al.* Advances in molecular quantum chemistry contained in the Q-Chem 4 program package. *Mol. Phys.* **113**, 184-215 (2015).

(11) Staroverov, V. N., Scuseria, G. E., Tao, J. & Perdew, J. P. Comparative assessment of a new nonempirical density functional: Molecules and hydrogen-bonded complexes. *J. Chem. Phys.* **121**, 11507 (2004).

(12) Furche, F. *et al.* Turbomole. *WIREs. Comput. Mol. Sci. 4*, 91-100 (2014).

(13) Weigend, F. & Ahlrichs, R. Balanced basis sets of split valence, triple zeta valence and quadruple zeta valence quality for H to Rn: Design and assessment of accuracy. *Phys. Chem. Chem. Phys.* **7**, 3297-3305 (2005).

(14) Grimme, S. Ehrlich, S. & Goerigk, L. Effect of the damping function in dispersion corrected density functional theory. *J. Comp. Chem.* **32**, 1456-1465 (2011).
(15) Kollwitz, M., Häser, M. & Gauss, J. Non-Abelian point group symmetry in direct second-order many-body perturbation theory calculations of NMR chemical shifts. *J. Chem. Phys.* **108**, 8295 (1998).
(16) Jusélius, J., Sundholm, D. & Gauss, J. Calculation of current densities using gauge-including atomic orbitals. *J. Chem. Phys.* **121**, 3952 (2004).
(17) Taubert, S., Sundholm, D. & Jusélius, J. Calculation of spin-current densities using gauge-including atomic orbitals. *J. Chem. Phys.* **134**, 054123 (2011).
(18) Fliegl, H., Taubert, S. Lehtonen O. & Sundholm, D. The gauge including magnetically induced current method. *Phys. Chem. Chem. Phys.* **13**, 20500-20518 (2011).
(19) Ahrens, J., Geveci, B. & Law, C. ParaView: *an end-user tool for large data visualization, visualization handbook* (Elsevier, 2005) ISBN-13: 978-0123875822, see also: http://www.paraview.org.
(20) Humphrey, W. Dalke, A. & Schulten, K. VMD: Visual molecular dynamics. *J. Mol. Graphics* **14**, 33-38 (1996).
