# Supplementary material for: Three-dimensional aromaticity in an antiaromatic cyclophane
Source: Nat Commun. 2019 Aug 8;10:3576. doi: 10.1038/s41467-019-11467-4 (PMC6687811; doi:10.1038/s41467-019-11467-4)
Supplement: Supplementary file 3 — Description of Additional Supplementary Files [file 41467_2019_11467_MOESM3_ESM.pdf]

Description of Additional Supplementary Files

File Name: Supplementary Movie 1
Description: Calculated current stream in the model system 5′ by the GIMIC method.

File Name: Supplementary Movie 2
Description: Calculated current stream in cyclophane 5 by the GIMIC method.
